# Seasonal variation in activity and nearshore habitat use of Lake Trout in a subarctic lake

**DOI:** 10.1186/s40462-023-00417-x

**Published:** 2023-08-31

**Authors:** Paul J. Blanchfield, Graydon McKee, Matthew M. Guzzo, Andrew J. Chapelsky, Peter A. Cott

**Affiliations:** 1 Freshwater Institute, Fisheries and Oceans Canada, Winnipeg, MB Canada; 2 Department of Biology, Queen’s University, Kingston, ON Canada; 3 Department of Biology, University of Toronto Mississauga, Mississauga, ON Canada; 4 Environment and Climate Change, Government of the Northwest Territories, Yellowknife, NT Canada

**Keywords:** Acoustic telemetry, Habitat coupling, Movement

## Abstract

**Background:**

In lake ecosystems, predatory fish can move and forage across both nearshore and offshore habitats. This coupling of sub-habitats, which is important in stabilizing lake food webs, has largely been assessed from a dietary perspective and has not included movement data. As such, empirical estimates of the seasonal dynamics of these coupling movements by fish are rarely quantified, especially for northern lakes. Here we collect fine-scale fish movement data on Lake Trout (*Salvelinus namaycush*), a predatory cold-water fish known to link nearshore and offshore habitats, to test for seasonal drivers of activity, habitat use and diet in a subarctic lake.

**Methods:**

We used an acoustic telemetry positioning array to track the depth and spatial movements of 43 Lake Trout in a subarctic lake over two years. From these data we estimated seasonal 50% home ranges, movements rates, tail beat activity, depth use, and nearshore habitat use. Additionally, we examined stomach contents to quantify seasonal diet. Data from water temperature and light loggers were used to monitor abiotic lake conditions and compare to telemetry data.

**Results:**

Lake Trout showed repeatable seasonal patterns of nearshore habitat use that peaked each spring and fall, were lower throughout the long winter, and least in summer when this habitat was above preferred temperatures. Stomach content data showed that Lake Trout acquired the most nearshore prey during the brief spring season, followed by fall, and winter, supporting telemetry results. Activity rates were highest in spring when feeding on invertebrates and least in summer when foraging offshore, presumably on large-bodied prey fish. High rates of nearshore activity in fall were associated with spawning. Nearshore habitat use was widespread and not localized to specific regions of the lake, although there was high overlap of winter nearshore core areas between years.

**Conclusions:**

We provide empirical demonstrations of the seasonal extent to which a mobile top predator links nearshore and offshore habitats in a subarctic lake. Our findings suggest that the nearshore is an important foraging area for Lake Trout for much of the year, and the role of this zone for feeding should be considered in addition to its traditional importance as spawning habitat.

**Supplementary Information:**

The online version contains supplementary material available at 10.1186/s40462-023-00417-x.

## Background

Mobile top predators can exert disproportionate influence on the ecosystems they inhabit because of their ability to consume resources within and across a variety of habitat types [1, 2]. Foraging across a variety of habitats, known as habitat coupling, is believed to stabilize food webs as predators can alter their forage base by moving to new areas as prey become depleted within habitats [3–5]. In freshwater lakes, the movement of generalist top predators amongst spatially discrete habitats allows for individuals to forage across nearshore (littoral), benthic, and offshore (pelagic) areas [2, 3, 6, 7]. While recognition of predator movement as a mechanism that links spatially discrete food webs within lakes has become more widely accepted in the past two decades since these ideas were initially presented [6–9], our understanding of the seasonal timing and extent of these connections remains limited.

The Lake Trout (*Salvelinus namaycush*), a cold-water stenotherm with a varied diet, has become a model for examining habitat coupling in lakes. In general, Lake Trout require cold (< 12 °C) well oxygenated (dissolved oxygen > 4 mg L^− 1^) water [10–15]. Many of the lakes that provide ideal oxythermal habitat for Lake Trout stratify in the summer months [16–18], forcing trout to occupy deeper, more offshore areas of the lake and thereby limiting opportunities for access to nearshore areas because of high water temperatures there [19–22]. Despite these thermal restrictions, numerous netting studies have captured Lake Trout in warm or shallow (< 6 m deep) areas of lakes in summer [23, 24]. Likewise, telemetry studies show that Lake Trout can occupy shallow, thermally suboptimal areas of lakes in summer [22, 25]. However, movements to shallow water are thought to be brief foraging forays that occur mainly in lakes lacking highly profitable cold-water prey (i.e. Class 1 lakes *sensu* [26, 27]).

To date, much of the evidence to support the use of littoral energy by Lake Trout comes from studies that have examined stomach contents or inferred diet from stable isotope analysis. Summer stomach content data show the presence of nearshore food items, such as nearshore fishes and benthic invertebrates, even in lakes with abundant cold-water pelagic prey fish, such as Cisco (*Coregonus artedi*) [27, 28]. Spatial differences in the carbon isotope values of the food web, with nearshore more enriched in δ^13^C compared to pelagic areas, have allowed for partitioning of the amount of energy fish acquire through littoral sources [29–31]. Lake Trout show greater littoral coupling in less reticulated (i.e. more circular) lakes [32] as well as in colder lakes [23, 33] because of the lower thermal stress associated with nearshore habitat use across the gradient of lakes examined. Likewise, Lake Trout trophic position, determined using stable isotopes of nitrogen (δ^15^N) [34], is lower in smaller lakes, suggesting a diet more reliant on invertebrates in these lakes because of limited access to forage fish [26, 27, 35, 36]. Despite abundant evidence for habitat coupling by Lake Trout, with both nearshore and pelagic food webs contributing to their diets, where and when Lake Trout acquire littoral energy still remains uncertain. In part this is because we have limited information on the seasonality of offshore movement by nearshore prey in boreal lakes [37, 38], but also because our understanding of the seasonal spatial ecology of Lake Trout is incomplete.

The Lake Trout is considered a northern species, having a latitudinal distribution situated in the Canadian Arctic and subarctic regions [39, 40]. In the northern part of their range, Lake Trout is important culturally, supporting subsistence fisheries, in addition to being sought after in both commercial and recreational fisheries. Yet, much of what we know about Lake Trout foraging ecology comes from lakes at the southern edge of this species’ distribution [41]. Strong lake stratification during summer is a common feature of these more southerly lakes and has shaped our understanding of habitat coupling by Lake Trout. For example, several studies have highlighted the importance of the spring season to Lake Trout growth in southern lakes [42], such that cool and prolonged spring periods (often defined as ice-off until surface waters warm to > 15 °C) favors growth irrespective of whether or not cold-water prey fish inhabit the lakes [22, 43]. Northern lakes, however, have much shorter periods of stratification, or none at all. Lake Trout in Great Bear Lake (66 °N), for example, rely heavily on nearshore prey in summer [44], and several shallow-water morphs exist within this lake that incorporate temporal pulses of terrestrial insects into their diet [45]. Summer catches of Lake Trout in nearshore areas have been shown to increase across a latitudinal gradient from south to north [23], and demonstrate the need for more northern-focused research on this species. Furthermore, studies in northern lakes on Lake Trout diet and habitat use have almost exclusively focused on the ice-free season, which may last only 2–3 months in some parts of the species range. This restricted sampling window fails to capture seasonality, which is an important aspect for understanding how Lake Trout may be impacted by warming or other regional stressors, including the intense pressure for increased resource development that often involves alteration to lakes or their watersheds [46–49].

In this study, we sought to quantify the seasonal movements of Lake Trout in a subarctic lake to examine their use of nearshore habitat, which we coupled with dietary data to examine reliance upon nearshore prey. We continually monitored the space- and depth-use of Lake Trout over a period of two years using spatial positioning acoustic telemetry that incorporated accelerometer transmitters to estimate activity. We specifically test for differences among seasons in activity and nearshore habitat use, both within and between years of study. With this approach, we ask whether there are specific areas of the nearshore zone that are preferentially visited by Lake Trout, and whether this changes seasonally. We also examine the influence of light on fish activity and habitat use during winter (ice cover) by comparing periods of complete darkness (early winter) versus light (late winter). Telemetry data is supported by in situ water temperature profiles, ice break-up and formation dates, lake bathymetry and habitat maps, as well as diet data collected from Lake Trout.

## Methods

### Study area

**Fig. 1:**
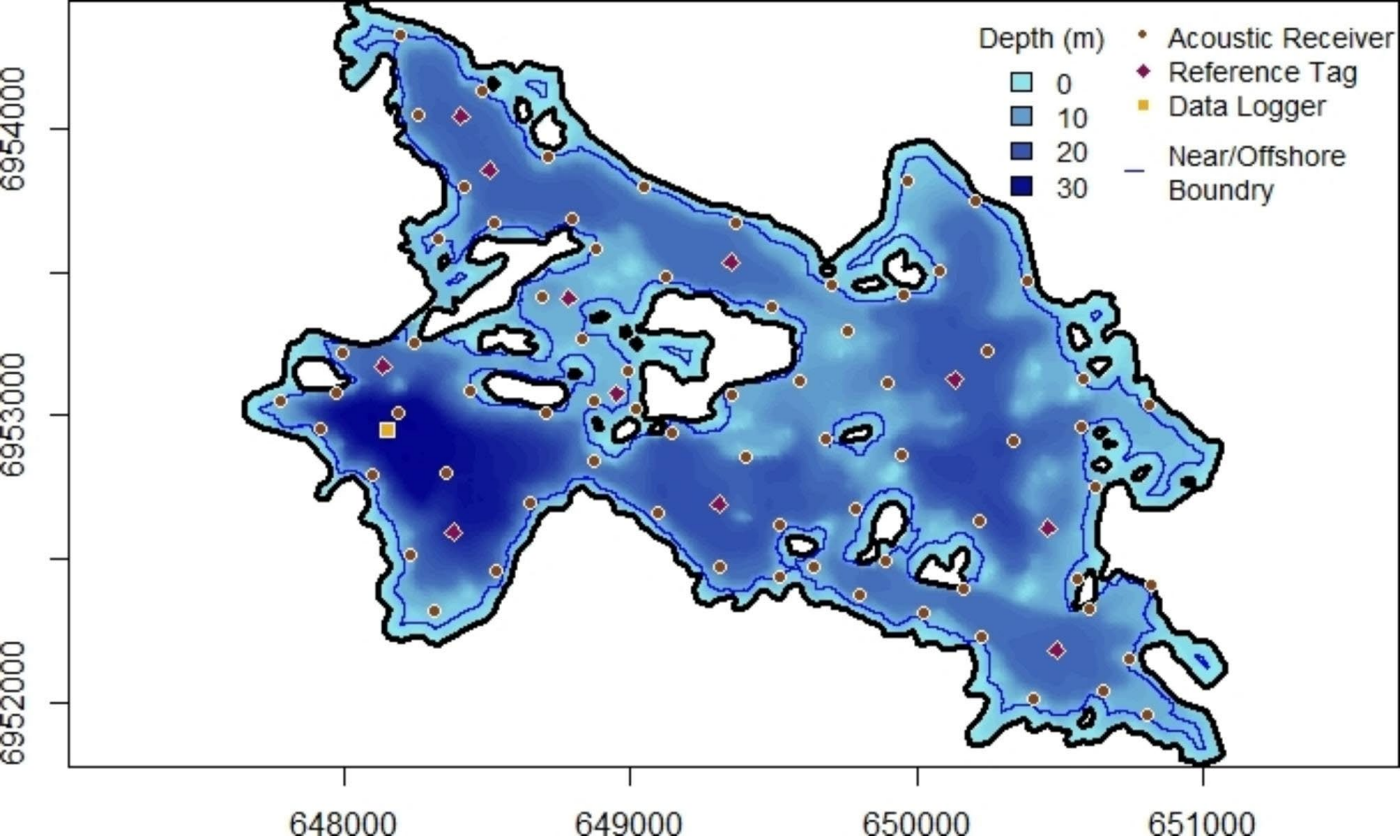
Bathymetric map of Alexie Lake with the location of acoustic receivers and associated sync tags, acoustic reference tags, the temperature and a light logger string, and the near/offshore boundary. The x- and y-axes show UTM coordinates in zone 11 N

This work took place at Alexie Lake, located about 30 km north-east of Yellowknife, Northwest Territories, Canada (62°40′36.59″ N, 114°4′22.76″W). Alexie Lake is an oligotrophic lake that thermally stratifies in summer, has a surface area of 402 ha, and reaches a maximum depth of 32 m (Fig. 1). A narrow channel in the northwest portion of Alexie Lake connects it to upstream Chitty Lake via a small basin. The fish community is comprised of three top level piscivores - Lake Trout, Northern Pike (*Esox lucius*), and Burbot (*Lota lota*) - as well as Lake Whitefish (*Coregonus clupeaformis*), Cisco, Lake chub (*Couesius plumbeus*), Ninespine Stickleback (*Pungitius pungitius*), Trout Perch (*Percopsis omiscomaycus*), Deepwater Sculpin (*Myoxocephalus thompsoni*), Slimy Sculpin (*Cottus cognatus*), and Spoonhead Sculpin (*Cottus ricie*) [47]. In addition to fish, Alexie Lake also contains *Mysis diluvania* and several other invertebrate species. Alexie Lake is part of the Chitty Lakes Research Area and is closed for recreational fishing year-round.

### Fish telemetry

Lake Trout tagging occurred in June of each year of the study. Fish were captured from Alexie Lake via angling and brought to shore in holding containers for surgical implantation of acoustic transmitters (2012: n = 30; 2013: n = 14). On shore, trout were anesthetized in a Tricaine Methanesulfonate (MS-222) and sodium bicarbonate buffer solution, and then intra-coelomically fitted with pressure-sensing (depth) acoustic transmitters (V13P-1; Vemco Ltd., Bedford, NS; random transmission intervals between 80 and 160 s) following standard surgical procedures [50]. Each depth sensor was calibrated from the surface of Alexie Lake to the maximum depth (32 m) at 4 m intervals and was accurate to ± 1.7 m with a 0.15 m resolution. The 14 trout tagged in 2013 were fitted with acoustic transmitters capable of measuring fish acceleration (V13AP-1 L, tail beat algorithm, 40 s sampling). The transmitters reported acceleration and pressure at different delay intervals during the study to extend battery life (80–160 s delay, mean delay = 120 s: June 15 - October 31, 2013 and June 1–30, 2014; 1000–1200 s delay, mean delay = 1100 s, November 1, 2013 - May 31, 2014). Acceleration measurements were converted to swimming speed following equations in Cruz-Font et al. [51].

This study made use of a Vemco Positioning System (VPS; Vemco Ltd.) consisting of 72 omnidirectional acoustic hydrophone-receivers (VR2W, 69 kHz) anchored to the bottom of Alexie Lake to track the depth and coordinates of acoustically tagged Lake Trout between June 2012-June 2014 (Fig. 1). Receivers were spaced based on detection range testing in Alexie Lake prior to this study such that detection ranges overlapped allowing for almost complete coverage of acoustically tagged Lake Trout within the lake. Internal clock synchronization was achieved during data processing with the aid of an acoustic transmitter (“sync tag”; V16-1 L, 69 kHz, random transmission intervals every 1100–1300 s) suspended 1–2 m above each acoustic receiver. Eleven reference tags were distributed throughout Alexie Lake to assess positioning accuracy and further aid in array synchronization. Brief gaps in data collection occurred during receiver downloads each spring and fall. A longer gap in data occurred in the spring of 2013 due to ice breakup interfering with the telemetry array.

Data from all detected Lake Trout tagged in this study were used until a transmitter failed or was shed, or an individual died or departed from the study system, at which point all successive locations for that individual were removed. In addition to this filtering, all fish locations that fell outside of the shoreline boundary, or depth range of Alexie Lake were removed prior to analysis. We observed greater positional error in nearshore areas of the lake and during winter, likely because of lower receiver coverage and reflections from ice cover, respectively (PJB, unpublish. data). Because filtering fish locations based on positional error (as in Guzzo et al. [48]) would have disproportionately removed data from nearshore areas and the winter season, and potentially biased our interpretation of fish habitat use, we retained all fish location data. We did, however, compare movement data derived from the telemetry array with data from accelerometer transmitters, the latter representing an independent measure of movement not affected by positional error, to examine system performance throughout an entire year (see below). Our final dataset contained 4,621,717 Lake Trout locations between June 2012 and June 2014.

The depth of acoustically tagged Lake Trout in Alexie Lake derived from pressure sensitive tags was averaged for each fish during each week of June 2012-June 2014. A grand mean of weekly Lake Trout depths was then calculated to describe average weekly Lake Trout depth in Alexie Lake. Similarly, a weekly grand mean of Lake Trout speed was calculated using both changes in position in the telemetry array over time (June 2012-June 2014; hereon referred to as “array speed”) as well as from accelerometer tags implanted in a subset of fish (June 2013-June 2014; hereon referred to as “accelerometer speed”; n = 14). To assess the relationship between Lake Trout array speed and accelerometer speed, we compared the daily grand means of the two metrics using linear regressions for the ice-free and ice-covered periods of June 2013-June 2014 using fish with accelerometer tags.

Nearshore habitat in Alexie Lake was defined as any region of the lake that was less than 6 m in depth, and within 53 m of shore (Fig. 1). This definition included the areas around islands, which are numerous in Alexie Lake. We chose this depth to be consistent with how other studies have defined the nearshore area across a latitudinal gradient of Lake Trout lakes [23] and because it is approximately where the thermocline sets up in summer in Alexie Lake. Lake trout were deemed as having used nearshore habitat when a VPS location estimate was found within the nearshore zone, and deemed as having used offshore habitat when a location estimate was found within the offshore zone. The weekly proportion of time spent in nearshore habitat by each fish was determined by the proportion of locations in the nearshore area of Alexie Lake (in relation to the total number of detections for that fish in the week) between June 2012-June 2014. Again, a grand mean of the proportion of nearshore detections was calculated to describe weekly nearshore use by the tagged population.

### Habitat modelling

Seasonal phenology between June 2012-June 2014 was based on the duration of ice cover and lake stratification in each year. The summer period, defined as the start to end of lake stratification when the average daily water temperature of the upper 6 m of Alexie Lake was ≥ 15 °C [22, 23], began on June 25, 2012 and June 22, 2013. Fall began when lake stratification ended (0–6 m strata < 15 °C) on September 10, 2012 and August 29, 2013. Complete ice cover on Alexie Lake, determined from trail camera photos of the lake [48], marked the start of the winter period (October 31, 2012 and November 9, 2013), and conversely, the termination of ice cover marked the start of the spring period (May 27, 2013 and May 29, 2014), which lasted until lake stratification began.

Hourly water temperature (°C) and light penetration (lux) were recorded continuously from June 2012-June 2014 using HOBO Pendant data loggers (64 k model UA-002-64, Onset Computer Co., Cape Cod, MA). Measurements were recorded in the deepest point of Alexie Lake at 0.5 m depth, 1 m intervals from 1 to 20 m depth, and at 25 and 30 m depths. Mean daily temperatures and illuminance at each logger were calculated, and spline interpolation was used to interpolate daily mean values at 0.1 m intervals. Dissolved oxygen was measured at 1 m intervals at the deepest point of the lake in the winter, spring, and fall in both 2013 and 2014.

Alexie Lake bathymetry, and substrate hardness, class, and complexity were characterized using high resolution acoustic sensing following systematic 25 m spaced parallel transects with a 120 kHz Simrad EK60 7.0° x 7.0° split beam echo-sounder system (Milne Technologies, Keene, ON). Substrate hardness was determined by the amplitude of the second sonar echo return, where softer substrates have a lower amplitude second echo due to greater absorption of the transmitted energy. Substrate class was assigned based on the backscatter of the primary echo, where smooth substrates such as smooth rock and compact sand result in a sharp increase and subsequent decline in echo amplitude, while rough substrates result in a slower decline in echo amplitude. Finally, substrate complexity was determined by calculating the total distinct substrate variance within a 60 m radius of 3 × 3 m cells within Alexie Lake. For each Lake Trout location data point, a substrate hardness, complexity, and class value were assigned based on the value of the point over which the trout was located at that time.

### Diet data

Lake Trout were angled from Alexie Lake for stomach content analysis in the winter of 2012 (n = 15), springs of 2012 and 2013 (n = 27), and fall of 2013 (n = 16). Summer sampling did not occur. Where possible individual fish were measured for fork length and weighed to the nearest half gram (fall: n = 11; winter: n = 15; spring: n = 8). Gastric lavage was used to empty the stomach contents of each Lake Trout. Prey items were identified to species for fish, and as either *Mysis* or invertebrates for other invertebrates, and were weighed to the nearest tenth of a gram.

### Statistical analyses

To understand the effect of temperature as a potential driver of Lake Trout depth, array speed, and nearshore habitat use, average weekly values for these three metrics were compared to average weekly temperature (°C) at 1 m depth during the two-year tracking period using a Generalized Additive Mixed Model (GAMM) including the year of study (year 1 or year 2) as a factor and accounting for the repeated measurements of individual fish. Average weekly Lake Trout depth, array speed, and nearshore habitat use were also compared to average weekly illuminance (lux) at 1 m depth during the ice-covered periods of June 2012-June2014, using year of study as a factor and accounting for the repeated measures of individual fish. The impacts of illuminance were modeled separately from temperature because of high concurvity between temperature and illuminance, and during winter only because light levels vary so greatly during northern winters (0-5391 lx at 1 m).

Seasonal changes in Lake Trout accelerometer speed in both the nearshore and offshore habitats were assessed using a two-factor ANOVA accounting for the repeated measures of individual fish during the period of June 2013-June 2014. To further test the hypothesis that light levels drive changes in Lake Trout activity during northern winters, we partitioned winter into two seasons for the analysis: dark winter (first day of winter until the day before illuminance at 1 m increases again to 1 lx) and light winter (the day that illuminance at 1 m increases to 1 lx to the end of winter). Significant results were followed by individual single-factor ANOVAs within either near/offshore groups or seasonal groups with a Bonferroni p-value correction. Significant values within single-factor ANOVAs with more than two categories were followed by Tukey’s honest significant difference (Tukey’s HSD) tests with Bonferroni p-value corrections to determine where pairwise differences occurred.

Two-factor ANOVAs were also used to compare seasonal changes (June 2012-June 2014) in usage of substrate hardness and complexity by Lake Trout, while accounting for repeated measures of individuals. Again, where significant results were found, single-factor ANOVAs within either nearshore/offshore groups, or seasonal groups, with Bonferroni p-value corrections were used to identify where differences occurred. Tukey’s HSD tests followed significant single-factor ANOVAs to determine where pairwise differences existed. The average proportion of detections associated with different substrate types during the different seasons in either the nearshore or offshore zones were compared visually to better understand changes in substrate hardness and complexity use.

Core (50% isopleth) home ranges for locations in the nearshore zone were created for all acoustically tagged Lake Trout combined using the *k*-nearest neighbor method of the Local Convex Hull estimator [52] for each season of each year of the study. Home ranges were calculated using 1000 nearshore locations randomly selected without replacement. In all seasons, with increasing number of locations selected, home range size leveled off at or before 1000 points indicating that home range estimates were accurate using this number of locations. Using an equal number of locations to create home ranges allowed for comparisons of size and overlap between seasons. We compared core home range sizes constructed for each season between years 1 and 2 of study, as well as between seasons within each year of study. For each seasonal mean core home range we determined 95% confidence intervals using the 1000 iterations of nearshore home range calculations. Where 95% confidence intervals did not overlap between seasons, home range sizes were considered significantly different. Nearshore seasonal home range overlap was also assessed between years 1 and 2 of this study, as well as sequentially between seasons following each other. Mean overlap of core home ranges, as well as 95% confidence intervals around means, were determined with 1000 iterations of overlap calculations.

Seasonal (fall, winter, spring) species composition of stomach contents were determined by the average mass of each taxon found in Lake Trout stomachs in proportion to the average total mass of prey found in Lake Trout stomachs. Seasonal total stomach content mass, and total stomach content mass in proportion to total fish mass were compared using single-factor ANOVAs. We also tested for the influence of lake trout fork length on the seasonal stomach contents using linear regression.

## Results

Of the 44 Lake Trout tagged for this study, all but one were detected on the telemetry array. Transmitter failure, fish departure from Alexie Lake, and fish mortality all occurred during the study. Twenty-eight of the 30 trout tagged in 2012 were followed through the first year, and 18 were monitored through the entire study. Of the 14 trout tagged in 2013, 11 were tracked until the end of the study.

**Fig. 2:**
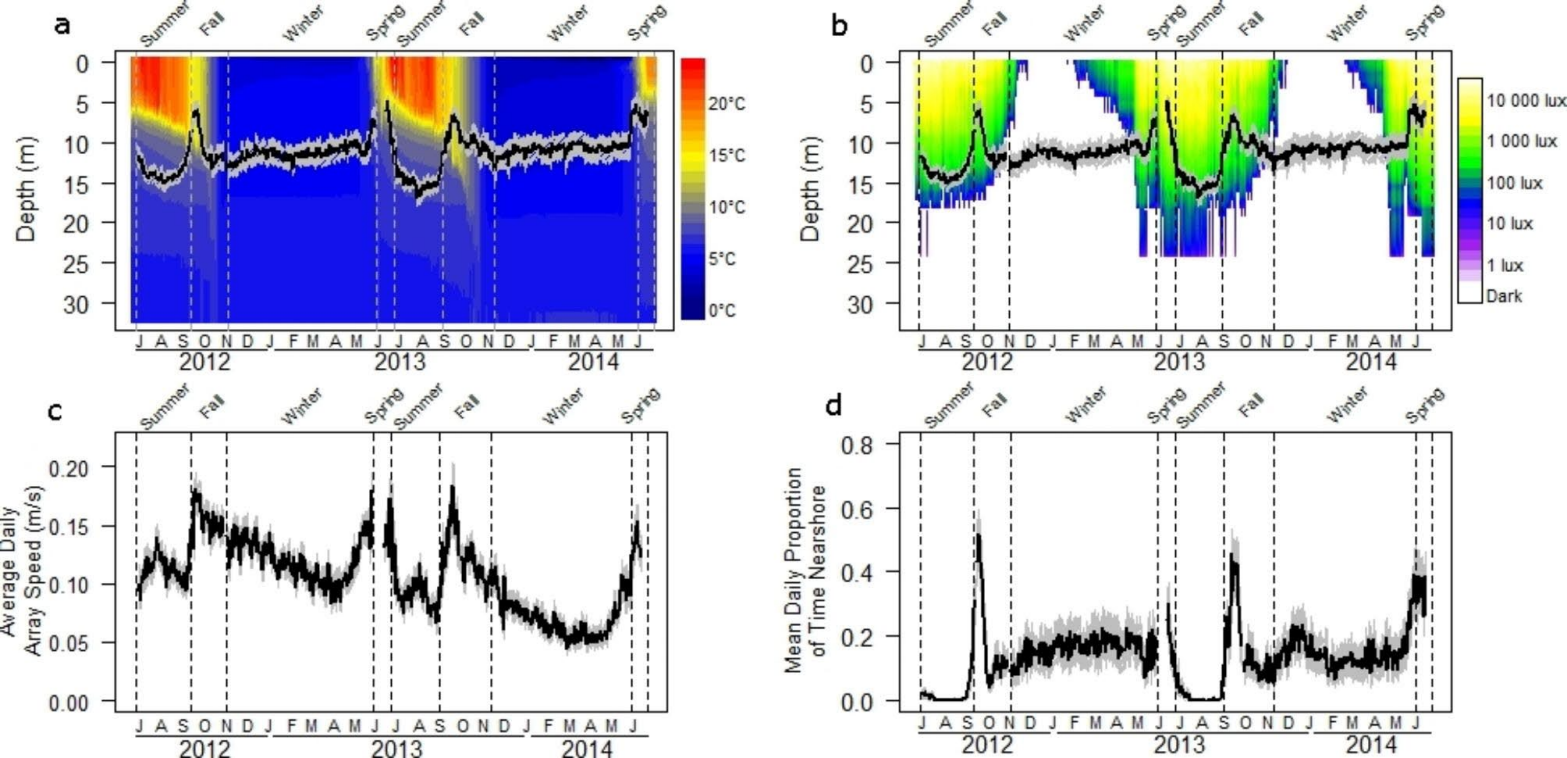
Average daily depth with standard error shading of acoustically tagged Lake Trout in Alexie Lake from June 2012-June 2014 overlaid on interpolated depth profiles of (**a**) water temperature and (**b**) log illuminance. Daily averages (with standard error shading) of (**c**) movement rates within the telemetry array and (**d**) proportion of time spent in the nearshore zone by acoustically tagged Lake Trout. Seasons are delineated by vertical dashed lines

The period of ice cover lasted almost 7 months at Alexie Lake, making winter the longest season (Fig. 2). Winter was followed by a brief spring period (< 1 month), with summer and fall lasting ~ 2.5 months and 2 months, respectively (Fig. 2). Surface water (1 m) temperatures in Alexie Lake ranged from a low of 0.6 °C in winter, to a high of 22 °C at the end of June and beginning of July (Fig. 2a). Surface light ranged from complete darkness in winter to 14 469 lx in June (Fig. 2b). Light penetration reached its deepest point of 25 m between May and October. Total dissolved oxygen less than 3 mg L^− 1^ occurred only at depths below 29 m in winter, decreasing to only the very bottom of the lake in spring, and then rising to 24 m in fall.

Depth, array speed, and nearshore habitat use of Lake Trout in Alexie Lake were all influenced by temperature. A generalized additive model indicated that in each year Lake Trout used the shallowest water when surface temperatures ranged between 12 and 15 °C (R²=0.70, yr 1: p < 0.001; yr 2: p < 0.001) corresponding to the spring and fall seasons (Fig. 2a). Further, the generalized additive model suggests that the changes in Lake Trout depth with temperature were similar between years (p = 0.647). The generalized additive models for Lake Trout array speed and proportion of time using nearshore habitat also indicated that these metrics peaked annually, each spring and fall, when surface temperatures ranged between 12 and 15 °C (array speed: R²=0.56, yr 1: p < 0.001; yr 2: p < 0.001, Fig. 2c; nearshore use: R²=0.57, yr1: p < 0.001; yr 2: p < 0.001, Fig. 2d). Like depth, the predictive models for array speed and nearshore use did not significantly differ between years (array speed: p = 0.991; nearshore use: p = 0.552).

During winter the impact of light levels on Lake Trout depth, array speed, and nearshore habitat use were less clear. Generalized additive models indicated significant trends between surface light levels and all metrics (depth: R²=0.85, yr 1: p < 0.001, yr 2: p < 0.001; array speed: R²=0.70, yr 1: p < 0.001, yr 2: p < 0.001; nearshore use: R²=0.78, yr 1: p < 0.001, yr 2: p < 0.001, Fig. 2) with increasing light levels leading to shallower depth occupancy, faster swimming speeds and greater nearshore use by Lake Trout. However, all smoother fits were nearly horizontal indicating that light levels did not have a strong influence on these metrics. Like temperature, the influence of winter light levels did not differ for depth or nearshore use between years (depth: p = 0.437; nearshore use: p = 0.75), but it did for swimming speed, with average trout array speeds generally being higher in the first year compared to the following year (p < 0.001; Fig. 2c). In each year, we observed a steady decline in Lake Trout movement rate as winter progressed (Fig. 2c).

**Fig. 3:**
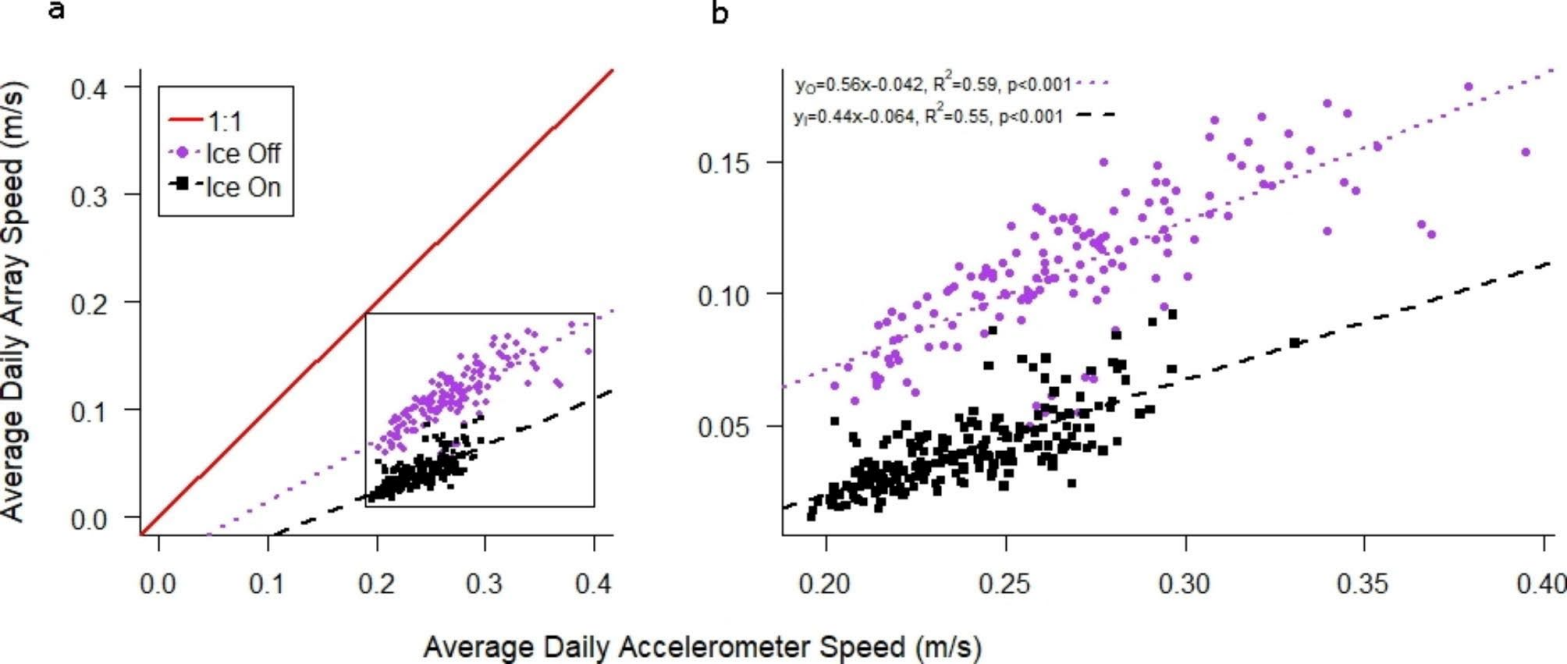
(**a**) Lake Trout average daily speed derived from spatial positions in the telemetry array deployed in Alexie Lake (see Fig. 1) were slower than average daily swim speed for those same fish measured using accelerometer tags. Data are from Lake Trout (n = 13) tagged with accelerometer transmitters during the period June 2013-June 2014. (**b**) Separate regressions are shown for the open-water season (ice-off; spring, summer and fall) and during the period of ice cover (ice-on; winter)

### Activity

Movement rates of Lake Trout in Alexie Lake determined from accelerometer transmitter data were consistently faster than those estimated based on spatial location data derived from the telemetry array (Fig. 3a). Average daily swimming speeds of Lake Trout using these different methods were positively and linearly correlated for both the ice present (winter: R²=0.55, p < 0.001), and open-water (spring, summer, and fall: R²=0.59, p < 0.001) periods (Fig. 3b). Both measures of Lake Trout swim speed (array and acceleration) showed a reduction during winter compared to the open-water period, although for a given acceleration speed the corresponding array speed was much lower in winter compared to the open-water seasons (Fig. 3b). We observed similar slope and variation (R^2^) for the relationships between array- and acceleration-derived swim speeds during the ice-covered versus open-water periods. However, because the accelerometer derived speeds are an independent measure of Lake Trout movement rate, and not subject to spatial and seasonal fluctuations in positional error, we preferentially use these data to provide estimates of movement rates where possible.

**Fig. 4:**
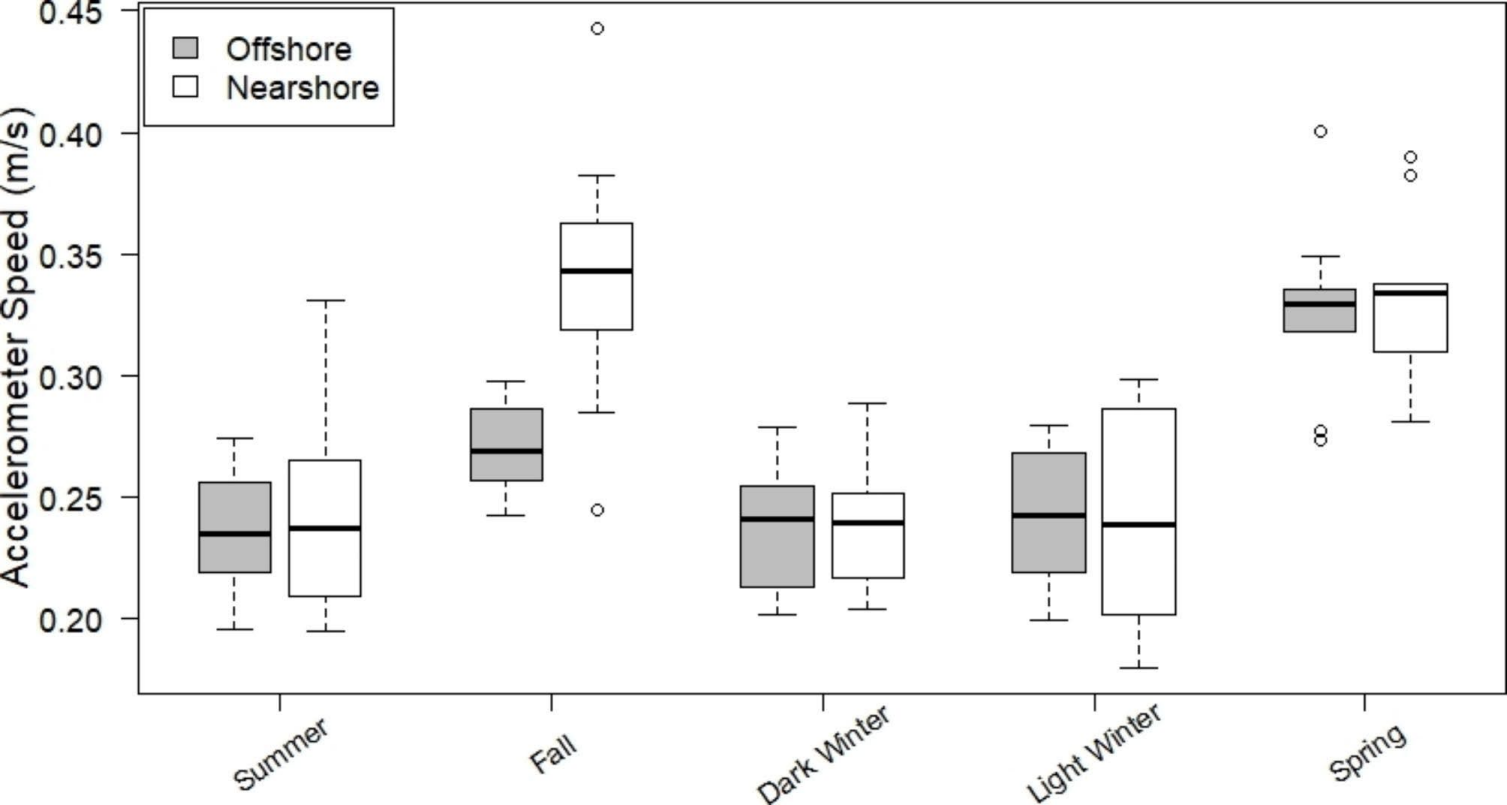
Seasonal movement rates of Lake Trout tagged with acceleration sensing acoustic tags in the offshore (shaded boxes) and nearshore (open boxes) zones of Alexie Lake from June 2013-June 2014 with median (black lines), interquartile range (boxes), 2.5 times the interquartile range (whiskers) and extreme values (points) shown

Lake Trout movement rates, based on acceleration values, were significantly influenced by both season and habitat (nearshore vs. offshore), as well as a significant interaction between season and habitat (two-factor ANOVA, season: p < 0.001; near/offshore use: p < 0.001; interaction: p < 0.001). Within a given season, Lake Trout movement rates differed significantly between nearshore and offshore zones only in the fall period (p_adj _= 0.0016), with accelerometer speeds being higher in the nearshore zone (Fig. 4). In no other season did accelerometer speed significantly differ between the nearshore and offshore habitats (p_adj _= 1).

Lake Trout movement rates varied significantly across seasons within both nearshore (p_adj _< 0.001) and offshore (p_adj _< 0.001) zones (Fig. 4). Summer and winter pairwise comparisons of accelerometer speed were not significantly different (Tukey’s HSD: p_adj _= 0.92-1). In the nearshore zone, fall and spring accelerometer speeds were significantly higher than summer and winter accelerometer speeds (p_adj _< 0.001), but did not significantly differ from each other (p_adj _= 0.99). In the offshore zone, accelerometer speeds were significantly higher in spring than all other seasons (p_adj _< 0.001). Movement rates in the offshore zone were significantly higher in fall than summer (p_adj _= 0.012), but fall accelerometer speeds did not significantly differ from the winter seasons in the offshore zone (p_adj _= 0.072–0.090).

### Habitat

**Fig. 5:**
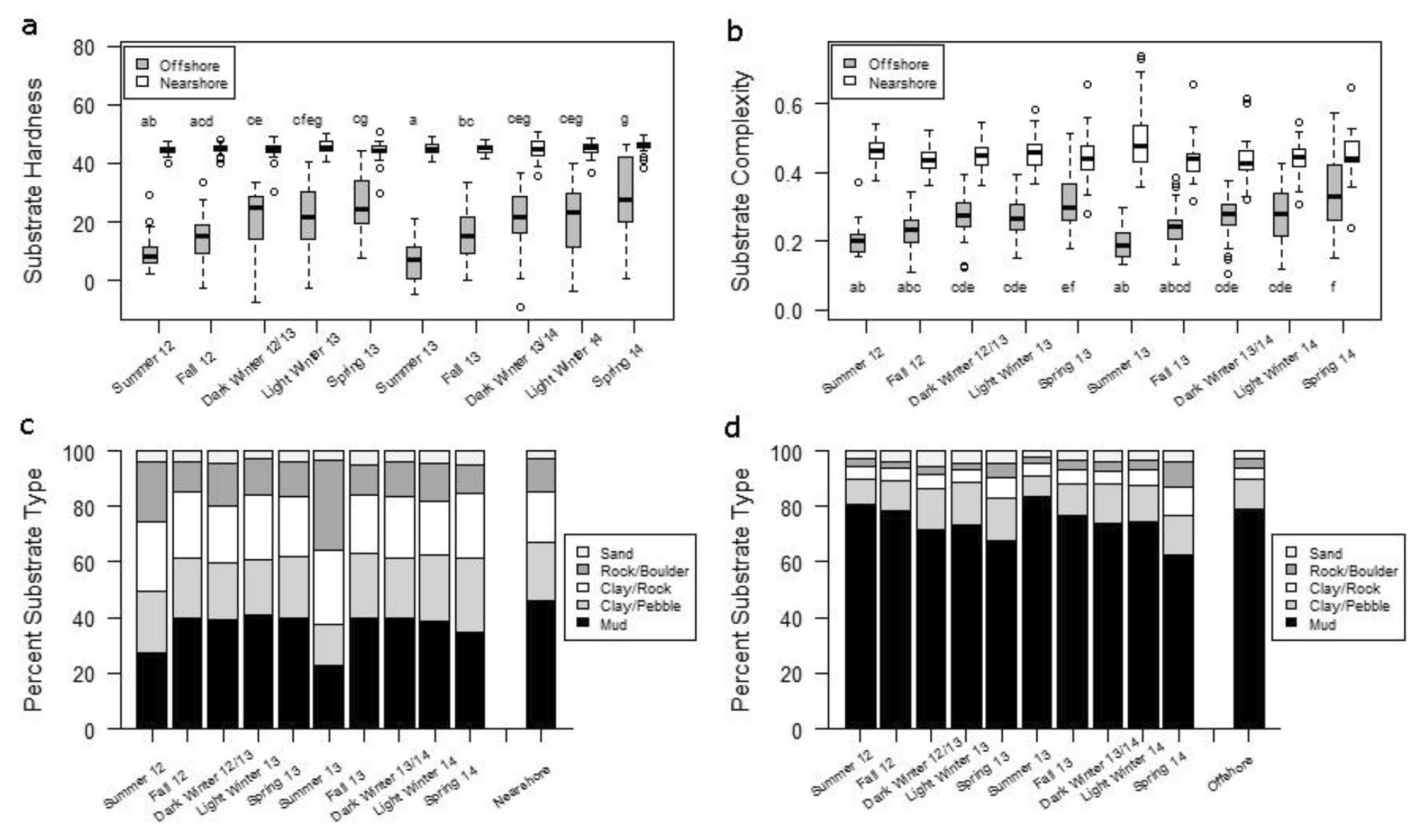
Seasonal substrate (**a**) hardness and (**b**) complexity based on spatial positions of acoustically tagged Lake Trout in the offshore and nearshore zones of Alexie Lake from June 2012-June 2014. Larger values indicate harder or more complex substrates, with median (black lines), interquartile range (boxes), 2.5 times the interquartile range (whiskers) and extreme values (points) shown. Letters indicate if significant differences exist between seasonal substrate hardness or complexity in the offshore zone, where seasons sharing the same letter do not significantly differ. Percentage of different substrate types over which acoustically tagged Lake Trout were positioned in the (**c**) nearshore zone and (**d**) offshore zone and the overall substrate composition for each zone

The nearshore zone of Alexie Lake constituted 29.5% of the total lake area (Fig. 1). Alexie Lake substrate hardness values ranged from − 15 to 65 and complexity values ranged from < 0.1–1.6, with larger values representing harder and more complex substrates, respectively. The dominant substrate type in the nearshore zone of Alexie Lake was mud (46%), although clay/pebbles (21%), clay/rock (18%), rock/boulder (12%), and sand (4%) substrates collectively contributed the most to the nearshore area (Fig. 5c). In contrast, the bottom substrate of the offshore zone of Alexie Lake was dominated by mud (79%), and consisted to a lesser degree of clay/pebbles (11%), clay/rock (4%), sand (3%), and rock/boulder (2%; Fig. 5d).

The bottom substrate hardness over which Lake Trout were positioned was significantly influenced by season (two-factor ANOVA: p < 0.001), habitat (nearshore vs. offshore; p < 0.001), and an interaction between season and habitat (p < 0.001). Within all seasons, Lake Trout were associated with harder substrates when nearshore than offshore (p < 0.001; Fig. 5a). When Lake Trout were present in the nearshore zone, there were no significant differences in substrate hardness among seasons (p = 0.30), whereas within offshore habitat Lake Trout were positioned over increasingly harder substrate as the seasons progressed from summer to spring, in a pattern that repeated itself in both years of the study (Fig. 5a).

Similar to bottom substrate hardness, the substrate complexity over which Lake Trout were positioned was significantly influenced by season (two-factor ANOVA: p < 0.001), habitat type (nearshore vs. offshore; p < 0.001), and an interaction between season and habitat (p < 0.001). Again, within all seasons, Lake Trout were associated with more complex habitat when nearshore than offshore (p < 0.001; Fig. 5b). Within habitat types, there was also seasonal variability in substrate complexity (nearshore: p = 0.04; offshore: p < 0.001). Mirroring substrate hardness, Lake Trout were present over increasingly complex substrates in the offshore zone of Alexie Lake as seasons progressed from summer to spring of the following year (Fig. 5b). In the nearshore zone Lake Trout substrate complexity use largely did not change significantly between seasons (p_adj _= 0.057-1), except for the summer of 2013 when Lake Trout were associated with slightly more complex habitat than the fall of 2012 (p_adj _= 0.005), fall of 2013 (p_adj _= 0.009), dark winter of 2013/14 (p_adj _= 0.011), and light winter of 2014 (p_adj _= 0.015; Fig. 5b).

Lake Trout in Alexie Lake were associated with similar substrate types in the nearshore zone throughout the fall, winter, and spring seasons but were less often associated with mud substrate in favour of rock, boulder, clay, and pebble during summer; a pattern that was consistent in both years of the study (Fig. 5c). In the offshore, Lake Trout primarily were detected over mud substrate, however there was a steady decline in the locations over mud substrates and greater representation of rock, boulder, clay, and pebble substrates as summer progressed to the following spring of each year (Fig. 5d).

### Nearshore habitat occupancy

Lake Trout presence in the nearshore zone was least in summer (< 2% of all detections) and much lower than at any other time of year (9–27%, Table 1; Fig. 2d). Core nearshore home range size (50% isopleth) was consistent through fall, winter, and spring, but much smaller during summer (Table 1; Fig. S1). Winter, spring, and summer core nearshore home range sizes were consistent between years; however, this was not the case for fall. Notably, during the first fall of study (2012), Lake Trout core nearshore home range size was far larger than those from any other season during the study period (Table 1).

**Table 1:** Mean (with 95% confidence intervals) of core (50% isopleth) nearshore home range areas for acoustically tagged Lake Trout during each season, starting in the summer of 2012 through spring of 2014 in Alexie Lake, NWT. The total number of spatial positions recorded in the nearshore area and the proportion of total positions this represents during each season is included

Season	Mean Core Area (m^2^)	Nearshore Positions	%Nearshore Positions
Summer (S1)	25,024 (20,158–30,822)	12,085	1.6
Fall (F1)	139,419 (110,827–169,598)	60,988	13.7
Winter (W1)	45,897 (37,994–55,572)	141,784	9.6
Spring (Sp1)	63,910 (51,630–78,170)	4,104	13.4
Summer (S2)	17,382 (14,079–21,542)	2,233	0.6
Fall (F2)	66,078 (47,890–86,608)	53,269	14.1
Winter (W2)	53,886 (44,534–63,313)	93,491	9.2
Spring (Sp2)	65,603 (46,712–86,393)	17,128	26.9

Overlap of Lake Trout core nearshore home ranges between years for a given season was highest in winter (60%, 95% CI = 48–71%), followed by fall (26%, 95% CI = 15–37%), summer (19%, 95% CI = 11–28%), and spring (15%, 95% CI = 5–27%). From one season to the next there was almost no overlap in core nearshore home range areas from winter to spring and from spring to summer, but more that 25% overlap was observed from summer to fall and ~ 15% overlap from fall to winter (Table 2; Fig. S1).

**Table 2:** Mean (with 95% confidence intervals) of core (50% isopleth) nearshore home range overlap of acoustically tagged Lake Trout in Alexie Lake between sequential seasons (earlier season/later season x 100%) from summer 2012 through to spring 2014

Season	Mean Overlap (%)	
	Year 1	Year 2
Spring to Summer	na	1.3 (0.0–5.4)
Summer to Fall	57.5 (39.3–75.6)	27.6 (11.8–39.0)
Fall to Winter	14.6 (6.5–21.3)	16.6 (1.9–28.7)
Winter to Spring	1.4 (0.0–9.4)	0.6 (0.0–6.8)

### Diet

Nearshore prey fish available to Lake Trout in Alexie Lake included Slimy Sculpin, Spoonhead Sculpin, and Ninespine Stickleback as well as young Northern Pike and Burbot, while offshore prey fish included Cisco, Deepwater Sculpin, smaller-bodied Lake Whitefish and Burbot, and young Lake Trout. *Mysis* were also captured in the offshore zone. Arthropods, Chironomid larvae, Daphnia, Dragonfly larvae, Finger Clams, Leeches, Mayfly larvae, Snails, and Caddis Fly larvae made up the nearshore invertebrate species available to Lake Trout.

**Fig. 6:**
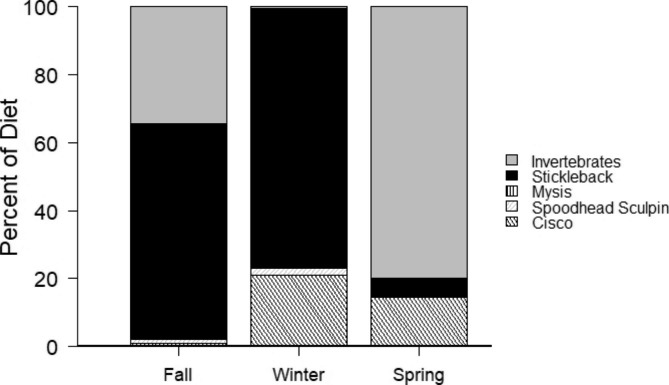
Seasonal diet composition of Lake Trout from Alexie Lake based on stomach content sampling in winter and spring of 2012 and in spring and fall of 2013

Lake Trout consumed similar amounts of prey in fall, winter, and spring in terms of total mass of prey consumed (p = 0.97) and total prey mass as a proportion of fish mass (p = 0.48). Stomach content analysis indicated that Lake Trout primarily forage on Ninespine Stickleback in the fall and winter, transitioning to heavy feeding on invertebrates in the spring (Fig. 6). Overall across all seasons we found no influence of Lake Trout fork length on the total mass of prey fish (β = 0.04 ± 0.03, p = 0.12) or invertebrates in their diets (β = 0.001 ± 0.007, p = 0.85). In winter, when Lake Trout ate almost exclusively prey fish, fork length did not predict the total mass of prey fish in their guts (β = 0.05 ± 0.06, p = 0.33). Similarly, in spring, when diets were dominated by invertebrates, Lake Trout fork length did not predict the total mass of invertebrates contained in their guts (β = 0.003 ± 0.01, p = 0.80). In fall, when diets were more mixed, Lake Trout fork length was not a predictor of the total amount of food found in their guts (β = 0.07 ± 0.06, p = 0.31).

## Discussion

We found that the nearshore area was an important component of Lake Trout habitat for much of the year in this subarctic lake. Apart from the relatively brief summer period (~ 2.5 months), when warm water temperatures limited access to the nearshore area, Lake Trout showed daily movement between offshore areas of the lake and the shallow nearshore region. Peak use of nearshore areas by Lake Trout occurred each spring and fall in a repeatable annual cycle that coincided with a narrow range of water temperatures (12–15 °C) and was accompanied by abrupt shifts in average depth and greater rates of movement compared to winter and summer seasons. Seasonal variation in Lake Trout spatial distribution was generally reflected in their diet, with greatest reliance on nearshore prey in spring and least in summer. Our findings provide one of the first empirical demonstrations of the extent of littoral-pelagic habitat coupling by a cold-water predator and the strong seasonality associated with nearshore habitat use in a subarctic lake.

The seasonal cycle of this subarctic lake was dominated by a long ice-covered period that lasted for more than half the year (~ 7 months), and for which there was complete lack of light penetration during much of the first half of winter. Lake Trout nearshore occupancy, however, was limited and largely stable over the entire winter before increasing as spring approached. Daily assessment of nearshore occupancy showed that Lake Trout spent only a small portion of time (~ 10%) in this region of the lake, and most time (~ 90%) in the offshore. Nearshore use by Lake Trout in small lakes lacking pelagic prey fish has been shown to be variable. In one study, Lake Trout appeared to predominantly occupy the upper few meters of the water column in offshore regions of the lake in winter [53], whereas in another study, Lake Trout were found to occupy both nearshore and offshore regions [54, 55]. In the present study, we expect that ice formation, which is typically much greater than in more southern areas and measured up to 75 cm thick in early winter [56], would have limited access to a portion of the nearshore area, and therefore winter use of this area is likely underestimated.

Winter nearshore home range core areas encompassed only ~ 4% of the nearshore area of Alexie Lake and was surprisingly consistent between years. Even more surprising was the high overlap (~ 60%) of these nearshore home range core areas from one winter to the next, which was more than twice that of the fall (26%); the season with the next greatest overlap and also the time of year when Lake Trout spawn in shallow areas of Alexie Lake [57]. Winter nearshore core areas were concentrated on the tips of islands and the southern shoreline surrounding the deepest basin of the lake, as well as off of islands and points in the shallower eastern portion of the lake (Fig. S1). Nearshore habitat types used in winter generally reflected that of the overall nearshore zone, indicating that these core areas of use were not atypical of this zone. Stomach contents revealed that Ninespine Sticklebacks were an important component of Lake Trout winter diet, while the telemetry data showed a strong offshore presence. Given the stability in water temperatures and low productivity during northern winters [58], Lake Trout are likely making use of forage from a variety of sources from all regions of the lake, a common strategy of opportunistic predatory fishes inhabiting nutrient-poor subarctic lakes. While we know little about the spatial distribution of prey fish in the study lake, the targeted use of certain areas by Lake Trout in Alexie Lake suggests that there may be highly profitable sites where nearshore prey, like Ninespine Stickleback, congregate during the winter months. This warrants further investigation.

In addition to lengthy ice-covered periods, another defining feature of northern regions is the dramatic seasonal change in day length [59]. In our study area, 24 h of twilight and daylight occurs for much of the summer period (late May to mid-July) declining to a minimum of ~ 7 h in late December [60]. Short day length coupled with extensive snow and ice cover resulted in extreme reduction in light penetration into Alexie Lake. This period, which we termed “dark winter”, lasted for roughly the first half of the winter period and was followed by high levels of light penetration through snow and ice cover in late winter (termed “light winter”) that were nearly equivalent to the summer period. Our hypothesis that Lake Trout swimming behaviour and habitat use would vary within the winter based on light levels was not strongly supported. Lake Trout accelerometer speed did not differ between the dark and light portions of winter in either the nearshore or offshore regions of Alexie Lake. While we did find significant trends indicating increases in Lake Trout activity and nearshore use, and decreasing average depth with increasing light levels, these trends were not strong. Light limitation has been proposed to explain Lake Trout use of shallower depths in winter [53], a pattern observed in another southern lake without pelagic prey fish [54], and also in Chitty Lake [60], which is adjacent to Alexie Lake and has the same food web [47]. Average depth of Lake Trout remained deep (~ 10 m) and was stable through much of ice-covered period in Alexie Lake, indicating that light did not play a strong role in structuring over-winter habitat use by Lake Trout.

Activity rates of Lake Trout showed a steady decline as winter progressed up until the final few weeks before ice breakup, when activity began to steadily increase. Reductions in daily rates of movement began shortly after peak activity in fall that coincides with spawning and reached some of the lowest levels observed for the year by late winter. The exception was the summer period, when activity rates were about the same as the least active period of winter. The generally lower rates of activity in winter compared to summer have been broadly observed for fishes [61]. For Lake Trout, however, there is growing evidence of relatively high rates of winter activity that can be similar to, or even greater than, during summer [53, 55]. A steady decline in activity as winter progresses, which we observed in both years of our study, has not been documented for Lake Trout before. McMeans et al. [54] observed a dramatic decline in activity following fall spawning, after which Lake Trout remained at a consistently low level for much of the winter. The steady decline in movement rate observed in Alexie Lake may be an adaptation to the long northern winters, where Lake Trout gradually reduce activity to conserve energy for a burst of spring feeding triggered by increasing light levels just prior to ice breakup. Differences between studies may also be related to the food web in each lake. Lake Trout in Alexie Lake have access to Cisco, which were absent in the other study lake [55], and therefore may have more energy reserves to sustain greater rates of movement in winter, albeit gradually declining as winter progresses. We also observed significantly lower rates of activity in the second winter of the study, which followed a prolonged fall period that lasted three weeks longer than in the previous year. Differences in Lake Trout winter movement rates were also observed between years in a similar study at a more southern lake [55], and may reflect the energetic constraints imposed by the demands of the preceding fall spawning season.

We anticipated that movement rates of Lake Trout based on estimates from successive positions in the telemetry array (array speed) could yield slower speeds than those calculated from the output of the accelerometer sensor within the fish (accelerometer speed). This is because the array speed is calculated from the straight-line distance between successive fish positions, which is a minimum estimate of the entire path travelled over that time period. Interestingly, for a given accelerometer speed, we observed a slower estimated array speed in winter versus the open-water periods. A suite of factors may be responsible for this observation. Certainly, fish behaviour can be different in winter. In a previous study, Lake Trout were observed to occupy smaller areas in winter versus summer while maintaining similar daily movement rates [53]. Also, greater vertical movement is possible in winter compared to the open water period, when Lake Trout occupy shallower areas of the lake (spring and fall) or are constrained to the hypolimnion in summer, as was observed in neighbouring Chitty Lake [60]. Higher rates of vertical movement would lead to reductions in the estimate of horizontal distance moved (array speed) for a given amount of effort (accelerometer speed). We also note that the equations developed to examine the relationship between swimming speed and accelerometer data were based on lab trials conducted at a single water temperature (12 °C;[51]) and should be recalibrated across a range of environmentally relevant temperatures that Lake Trout occupy. Our estimates of average winter swim speed from the array (winter1: ~7.5 m min^− 1^; winter2: ~4.5 m min^− 1^) were comparable to, although slightly higher than, those for Lake Trout from a similar telemetry study in a southern lake, and which also demonstrated similar variation in swim speed from one winter to the next (winter1: 3.0 m min^− 1^; winter2: 5.8 m min^− 1^) [55].

Spring is a time of rapid warming in this subarctic region. In a span of just over three weeks, Alexie Lake went from being ice covered to water temperatures in the nearshore region (the upper 6 m of the water column) warming to 15 °C, a threshold temperature typically avoided by Lake Trout [21, 22]. During this brief spring period, Lake Trout swim speeds and use of the nearshore zone were high and similar to the fall spawning period. Nearshore positions were greatest in the second spring (27%), and twice that of the previous spring, although nearshore core home ranges were similar between years, occupying about 6% of the nearshore zone each spring. Nearshore use may have actually been higher in the first spring, but at this time we experienced a gap in data collection due to ice breakup interfering with the telemetry array. Most nearshore core areas were situated adjacent to islands and typically surrounded by shallower flats, although core areas were also positioned along the southern shore of the lake (Fig. S1). Greater association with more complex and harder substrates by Lake Trout each spring in the offshore region is consistent with the location of nearshore core areas. Because hard and complex substrate in Alexie Lake is concentrated in and around the nearshore regions of the lake, this finding indicates that when offshore Lake Trout are in the shallower areas at the edges of nearshore habitat, as opposed to being adjacent to steep drop-offs.

Lake Trout diet, based on stomach contents, shifted dramatically from winter to spring and became dominated by invertebrates. Littoral energy sources have been shown to be the dominant energy pathway to a closely-related salmonid, Arctic Char (*Salvelinus alpinus*), contributing 62–94% of total energy to this top predator in subarctic lakes [62]. Moreover, contributions of littoral energy to Arctic Char diet does not show seasonal peaks [63]. In contrast, the marked seasonality in acquisition of nearshore prey by Lake Trout, characteristic of more southern lakes and largely driven by thermal avoidance of nearshore areas in summer, was also present in this subarctic lake [22, 28, 55]. In Alexie Lake, consumed prey mass was similar across the open-water seasons. In lakes without pelagic prey fish Lake Trout stomachs tended to most full in spring (or were less likely to be empty), suggesting a gorging on nearshore prey during this brief seasonal window [22, 55]. Previous studies have shown that when Lake Trout do have access to offshore prey fish, spring dependence on nearshore prey is more limited or non-existent [55, 64]. Here we show that in this subarctic lake where Cisco are present, spring feeding on nearshore macroinvertebrates is an important seasonal component of Lake Trout foraging ecology.

Springtime activity was some of the highest observed for Lake Trout when comparing across all seasons. We interpret the high activity rates in spring as a strategy by Lake Trout to find and consume the large amount of invertebrate forage available during the brief window of thermal accessibility. For other lake-dwelling salmonids, higher rates of movement allow for greater encounter rates with planktonic forage [65]. In a previous telemetry study using accelerometers, Lake Trout activity was greater for populations feeding on planktivorous prey versus populations feeding on large-bodied prey fish [66]. This finding is consistent with modelling approaches demonstrating the increased activity required to acquire many smaller prey items (i.e. zooplankton) compared to an equivalent food amount from a larger prey item (i.e. Cisco) for different Lake Trout populations [67]. The high rates of activity in spring and low rates of activity in summer, when Lake Trout diet was dominated by the smallest (i.e. invertebrates) and presumably the largest (i.e. Cisco) individual prey items, respectively, provides further empirical support for these earlier findings. Importantly, our findings highlight how in a subarctic lake, seasonally available resources greatly influence dietary breadth, habitat coupling and foraging costs of a cold-water top predator.

Lake Trout were predominantly in the offshore region in summer, when nearshore areas were > 15 °C. This water temperature acts as a thermal deterrent, not present during other seasons, and limits nearshore habitat use by Lake Trout [22]. Lake Trout activity rates were low, and fish were located at deeper depths (~ 15 m) in summer than at other times of the year. Although only a small percentage of locations (~ 10%) were in the nearshore zone, and nearshore core home ranges were smallest (~ 2% of nearshore area) at this time, Lake Trout appeared to be selective in their habitat use. Specifically, when in the nearshore zone, fish occupied areas with harder substrates (i.e. clay, pebble, rock, boulder, sand) and were less associated with soft substrate (i.e. mud); a pattern that was observed in both summer periods and indicative of non-random habitat selection. Surprisingly, shallow habitat selected in summer was mostly associated with islands, either in the central portion of the lake or elsewhere, where nearby bathymetry was gradual (Fig. S1). We were not able to collect stomach content samples at this time of year, so we are unsure whether Lake Trout were targeting a specific prey item in these nearshore habitats. Likewise, we do not have direct evidence of the food items taken by Lake Trout in summer, but given their mostly offshore presence during this season we expect that they were primarily consuming Cisco, the dominant offshore prey in Alexie Lake [47], as has been found in other studies where offshore fish are available [28, 55].

Lake Trout spawning typically occurs in the nearshore areas of lakes in the fall [24]. At Alexie Lake, this coincided with the presence of Lake Trout in nearshore areas where they exhibited high rates of movement. Consistent with this general description of spawning activity, fall was the only season for which spatial variation in Lake Trout speed was evident, with average speeds being greater in the nearshore zone than offshore. Lake Trout in Alexie Lake spawn at water depths of ~ 2 m on cobble shoals that are spatially distributed around the lake [56, 57]. Despite this association with cobble substrate during spawning, we did not find evidence that this habitat was specifically selected during the fall season. This may have been because the cobble habitat used for spawning was interspersed among other habitat types, wide-ranging movements by Lake Trout in the nearshore traversed all habitat types during spawning, and that the fall period, defined by water temperature to when ice formed on the lake, included periods when Lake Trout were no longer spawning [56, 57]. While the daily proportion of nearshore positions was similar each fall (14%), nearshore core home range area was twice as large in the first year of the study, when the fall period was shorter (by 3 weeks) compared to the following year. For closely related Brook Trout (*Salvelinus fontinalis*), spawning season duration has been shown to be influenced by rates of cooling in the fall, with more rapid cooling leading to condensed annual reproductive activities [68]. However, why Lake Trout would have larger nearshore core home range areas during a more truncated spawning season is not entirely clear at this time. Also of interest is the finding that core nearshore home ranges in fall and summer overlapped to a greater degree than any other pair of seasons, even though the nearshore use by Lake Trout during the summer was extremely limited, leaving one to speculate that perhaps nearshore habitat use in summer also serves as a reconnaissance opportunity to assess spawning areas.

The fall season is one in which Lake Trout have often been considered as fully occupied with reproductive activities. In Alexie Lake, we show that Lake Trout are also actively feeding during the fall, with stomach fullness similar to other seasons. Fall diet showed the greatest reliance on nearshore prey and included Ninespine Sticklebacks as well as littoral invertebrates. This finding, however, runs counter to observations from other studies. For Arctic Char in subarctic lakes there is evidence of reduced reliance on littoral energy during the fall period [63]. Likewise, fall feeding by Lake Trout in more southern lakes can be diminished [64] or is dominated by offshore planktonic prey, such as Mysids [22]. It is also worthwhile to point out that skip spawning by a portion of the adult population can be more prevalent at northern latitudes. Records from our study lake show Lake Trout regularly skip spawning (PAC, unpublish. data). Thus, our findings of extensive opportunistic feeding by Lake Trout in the fall extend the seasonal perspective over which we consider habitat coupling to be important and may reflect a response to the greater energetic demands of a subarctic environment.

So far, we have discussed habitat coupling by Lake Trout without considering the other top predators that exist in the lake. In subarctic lakes, the presence of competing species can result in narrow niche widths that have the potential to reduce littoral-pelagic habitat coupling by salmonids [69]. Previously, we have examined habitat overlap among Lake Trout, Burbot, and Northern Pike during summer in Alexie Lake, and demonstrated that there was limited species interactions during this season [48]. While Lake Trout are predominantly found offshore and pelagic in summer, here we show they have much greater use of nearshore habitat in all other seasons. Notably, Alexie Lake contains large Northern Pike (> 10 kg) that are capable of consuming the size of Lake Trout we implanted with transmitters in this study (~ 1 kg). We observed numerous Lake Trout with scars indicative of predation attempts, and witnessed several attacks by large Northern Pike on Lake Trout during capture by angling (PJB, MMG, AJC, PAC, pers. obs.). These observations suggest that Lake Trout nearshore use, while frequent in Alexie Lake, may in fact be less than realized because of the threat of predation from Northern Pike in this system.

Subarctic and Arctic regions are experiencing some of the most rapid rates of warming on the planet [70]. This is resulting in significant changes to northern lake ecosystems, such as longer ice-free seasons, warmer lake surface temperatures, and increased lake productivity, to name a few [71]. The impacts of climate change on northern fish fauna is not well understood, in part due to the limited information available for this region [72]. In high-latitude lakes where Lake Trout reside, climate warming may increase available habitat and production within lakes, as well as open up new areas to colonize [73–75]. For example, Lake Trout growth has been shown to be positively correlated to mean August temperatures in some northern lakes [76]; however, a key uncertainty is whether the increased energetic demands of fish in a warmer environment can be met with projected increases in lake production [77, 78]. This uncertainty is exacerbated by the limited information available on seasonal habitat use and diet of northern Lake Trout populations and specifically the role of nearshore areas, which can disproportionately contribute to fish growth [22, 79] and are subject to the greatest change from a warming climate. Here we demonstrate that the nearshore area is important foraging habitat for Lake Trout for much of the year (fall, winter, and spring) and therefore reduced access to this portion of the lake from warming may adversely impact northern populations.

## Conclusion

Monitoring the spatial distribution of Lake Trout on a daily basis over a 2-year period demonstrated the true extent to which this top predator links littoral and pelagic habitats in a subarctic lake. Daily movements between nearshore and offshore regions of the lake occurred in all seasons, but less so during the summer period (~ 2.5 months) when Lake Trout were predominantly offshore. Littoral prey (Ninespine Sticklebacks and invertebrates) were dominant in the stomachs of Lake Trout in winter, spring, and fall, highlighting the importance of nearshore habitat during these seasons. Moreover, we found little annual overlap between core nearshore home ranges among seasons, indicating that the nearshore resources used by Lake Trout during these seasons are not fixed to a particular area, a finding worth noting when considering development projects which may impact nearshore habitats.

### Electronic supplementary material

Below is the link to the electronic supplementary material.

Supplementary Material 1

## Data Availability

The datasets analyzed during the current study are available from the corresponding author on reasonable request.
