# Supplementary material for: Seasonal variation in activity and nearshore habitat use of Lake Trout in a subarctic lake
Source: Mov Ecol. 2023 Aug 31;11:54. doi: 10.1186/s40462-023-00417-x (PMC10468872; doi:10.1186/s40462-023-00417-x)
Supplement: Supplementary file 1 — Supplementary Material 1 [file 40462_2023_417_MOESM1_ESM.docx]

**Seasonal variation in activity and nearshore habitat use of Lake Trout in a subarctic lake**

Paul J. Blanchfield^1,2*^, Graydon McKee^1^, Matthew M. Guzzo^3^, Andrew J. Chapelsky^1^, Peter A. Cott^4^

**Supplemental Information**

SI Figures


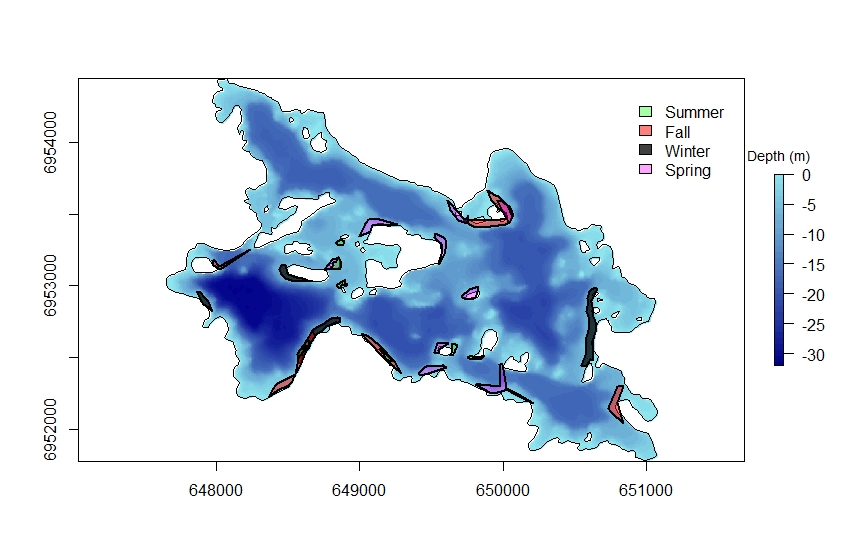


**Fig. S1** Seasonal nearshore core home range areas (50% isopleth) of acoustically tagged Lake Trout in Alexie Lake
